# Roles of the Sodium-Translocating NADH:Quinone Oxidoreductase (Na^+^-NQR) on *Vibrio cholerae* Metabolism, Motility and Osmotic Stress Resistance

**DOI:** 10.1371/journal.pone.0097083

**Published:** 2014-05-08

**Authors:** Yusuke Minato, Sara R. Fassio, Jay S. Kirkwood, Petra Halang, Matthew J. Quinn, Wyatt J. Faulkner, Alisha M. Aagesen, Julia Steuber, Jan F. Stevens, Claudia C. Häse

**Affiliations:** 1 Department of Biomedical Sciences, College of Veterinary Medicine, Oregon State University, Corvallis, Oregon, United States of America; 2 Department of Microbiology, College of Science, Oregon State University, Corvallis, Oregon, United States of America; 3 Linus Pauling Institute, Oregon State University, Corvallis, Oregon, United States of America; 4 Department of Pharmaceutical Sciences, Oregon State University, Corvallis, Oregon, United States of America; 5 Institute of Microbiology, University of Hohenheim, Stuttgart, Germany; University of Alberta, Canada

## Abstract

The Na^+^ translocating NADH:quinone oxidoreductase (Na^+^-NQR) is a unique respiratory enzyme catalyzing the electron transfer from NADH to quinone coupled with the translocation of sodium ions across the membrane. Typically, *Vibrio* spp., including *Vibrio cholerae*, have this enzyme but lack the proton-pumping NADH:ubiquinone oxidoreductase (Complex I). Thus, Na^+^-NQR should significantly contribute to multiple aspects of *V. cholerae* physiology; however, no detailed characterization of this aspect has been reported so far. In this study, we broadly investigated the effects of loss of Na^+^-NQR on *V. cholerae* physiology by using Phenotype Microarray (Biolog), transcriptome and metabolomics analyses. We found that the *V. cholerae* Δ*nqrA-F* mutant showed multiple defects in metabolism detected by Phenotype Microarray. Transcriptome analysis revealed that the *V. cholerae* Δ*nqrA-F* mutant up-regulates 31 genes and down-regulates 55 genes in both early and mid-growth phases. The most up-regulated genes included the *cadA* and *cadB* genes, encoding a lysine decarboxylase and a lysine/cadaverine antiporter, respectively. Increased CadAB activity was further suggested by the metabolomics analysis. The down-regulated genes include sialic acid catabolism genes. Metabolomic analysis also suggested increased reductive pathway of TCA cycle and decreased purine metabolism in the *V. cholerae* Δ*nqrA-F* mutant. Lack of Na^+^-NQR did not affect any of the Na^+^ pumping-related phenotypes of *V. cholerae* suggesting that other secondary Na^+^ pump(s) can compensate for Na^+^ pumping activity of Na^+^-NQR. Overall, our study provides important insights into the contribution of Na^+^-NQR to *V. cholerae* physiology.

## Introduction

Na^+^-translocating NADH:quinone oxidoreductases (Na^+^-NQR) are found in the respiratory chains of a number of marine and pathogenic bacteria (reviewed in [1], [2]). In organisms that express Na^+^-NQR, this enzyme is the gateway for electrons into the respiratory chain. Na^+^-NQR accepts reducing equivalents from NADH and donates them to the quinone pool [3], [4]. The energy from this redox reaction is used to pump sodium ions from the inner to the outer side of the membrane, thereby building a sodium motive force (SMF).

Besides Na^+^-NQR, two more types of respiratory NADH-dehydrogenases have been reported, the proton-pumping NADH dehydrogenases (NDH-1 or Complex I) and NDH-2 [5]. Similar to Na^+^-NQR, NDH-1 utilizes energy from the redox reaction, but it pumps protons from the inner to the outer side of the membrane to build a proton motive force (PMF). Unlike Na^+^-NQR and NDH-1, NDH-2 has neither H^+^ nor Na^+^ efflux activities. A few pathogenic bacteria, such as *Yersinia pestis* and *Pseudomonas aeruginosa*, have all three types of NADH dehydrogenases, but most bacteria possess one or two of these NADH dehydrogenases [5], [6].

Lack of the major NADH dehydrogenase activity usually significantly affect bacterial physiology. *Escherichia coli* has a single NDH-1 (encoded by *nuoA*-*N*) and NDH-2 (encoded by *ndh*), and it is known that NDH-2 is the major enzyme for aerobic growth, whereas the NDH-1 is essential for anaerobic fumarate and DMSO respiration [7]. Moreover, it is known that the *E. coli* strain lacking NDH-1 showed growth retardation and increased acetate secretion after the transition to stationary growth phase when grown in mixed amino acids media [8]. It was also shown that the *E. coli* strain lacking NDH-1 grew poorly when grown in minimal media supplemented with acetate as the sole carbon source [8].

*Vibrio cholerae* is the causative agent of cholera, a waterborne severe diarrheal disease. Genome sequencing revealed that *V. cholerae* does not have NDH-1 but has Na^+^-NQR (encoded by *nqrA-F*) and NDH-2 (encoded by *ndh*) [2], [6]. Although Na^+^-NQR is not essential for *V. cholerae* growth [9], the Δ*nqrA-F* mutant strain of *V. cholerae* shows a marked growth defect when grown in LB [10], similar to the *E. coli* strain that lacks a functional NDH-1 [8]. In addition, the *V. cholerae* Δ*nqr A-F* mutant strain shows multiple defects in carbon metabolism [10]. Furthermore, Na^+^-NQR also affects *V. cholerae* virulence gene expression possibly by affecting acetyl-CoA metabolism via the TCA cycle [9], [10] and survival *in vivo* [11].

In this study, we aimed to investigate the effects of a Δ*nqrA-F* mutation on broad aspects of *V. cholerae* physiology by using Phenotype Microarray (Biolog), transcriptome, and metabolomics analyses. We found that Δ*nqrA-F* mutant strain showed multiple defects in metabolism but did not show any defect in Na^+^ pumping-related phenotypes of *V. cholerae*.

## Materials and Methods

### Bacterial strains and growth conditions

*V. cholerae* O395N1, *V. cholerae* O395N1 Δ*nqrA-F* [12] and *V. cholerae* O395N1 Δ*pomAB* [13] strains were used in this study. Bacterial strains were kept at −80°C in 20% glycerol stocks. Bacterial strains were grown in Luria-Bertani (LB) medium at 30°C or 37°C. Streptomycin was supplemented at 100 µg/ml. When necessary, 33 mM L-lactate was used.

### Phenotype microarray (PM) analyses

Phenotype microarrays were performed using PM3 to PM10 MicroPlate (Biolog) at Biolog's PM Services group. All plates used pyruvate as sole carbon sources.

### DNA microarray analyses

Cells of *V. cholerae* O395N1 and *V. cholerae* O395N1 Δ*nqrA-F* strains, grown in LB (pH 6.5) at 30°C for 4 hours and 8 hours, were treated with RNA Protect Bacteria Reagent (Qiagen). RNA was extracted using the QIAGEN RNeasy Mini Kit (Qiagen). RNA was concentrated using the QIAGEN RNA MiniElute Cleanup kit (Qiagen) and sent to the Center for Genome Research and Biocomputing at Oregon State University, Corvallis, OR, following protocols outlined in the NimbleGen array user's guide, version 5 for cDNA synthesis and microarray analysis using NimbleGen Microarray plates and reagents. The microarray data are accessible at the Gene Expression Omnibus (accession number, GSE56387).

### Metabolite extraction

*V. cholerae* O395N1 and *V. cholerae* O395N1 Δ*nqrA-F* strains were grown in LB (pH 6.5) at 30°C for 2 hours and the supernatant was removed by centrifugation (8000 rpm, 4 °C, 5 min). The pellet was resuspended in 600 µL of LC-MS-grade water in 1 mM HEPES and 1 mM EDTA (pH 7.2). Metabolites were extracted from bacterial cells by using the boiling water method as previously described [14].

### LC-MS/MS

Mass spectrometry based metabolomic profiling was performed as previously described [15]. Briefly, liquid chromatography (LC) was performed on a Shimadzu Nexera system and metabolites separated on an Inertsil phenyl-3 stationary phase (GL Sciences, 5 uM, 4.6 × 150 mm). Mass spectrometry was performed on an AB SCIEX Triple TOF 5600 quadrupole-time-of-flight mass spectrometer. MS/MS spectra were gathered on the fly by information dependent acquisition. Most metabolites were identified by mass, isotope distribution, MS/MS fragmentation, and when standards were available, retention time. In the absence of chemical standards, MS/MS spectra were compared to those in the METLIN online database.

To account for analytical and sample preparation variation, samples were normalized to total ion count. Central energy metabolites (metabolites of the tricarboxylic acid cycle, pentose phosphate pathway, and glycolysis and amino acids) were targeted post-data acquisition and in addition, untargeted statistical analysis (Student's t-test p-value plotted against fold-change) revealed large (>10-fold) changes in cyclic AMP and cyclic GMP, and subsequently, related metabolites (purines and pyrimidines) were targeted post-data acquisition.

### Growth measurement

Growth measurement assays were performed as previously described [16]. Briefly, overnight grown bacterial cells were inoculated into 200 µl liquid medium in 96-deep-well plates (Whatman) at an initial OD600 of 0.05 and grown at 37 °C for 18 h with vigorous aeration. LBB medium (non-cationic L broth) was supplemented with streptomycin and varying concentrations of ethylene glycol. The initial pH of the media was adjusted with HCl to 6.5 and buffered by the addition of 60 mM BTP. Growth was then measured as OD600 by scanning the plates on a Bio-Rad iMark microplate absorbance reader. All experiments were repeated at least three times in triplicate.

### Measurement of acetate excretion

Acetate excretions from bacterial cells were measured as previously described [10].

### Motility assay

Motility of *V. cholerae* strains was determined on soft agar plates buffered with 100 mM Bis/Tris (pH 6.5) containing 100 mM NaCl, 0.25% agar, 1% tryptone, 0.5% yeast extract and 50 µg ml^−1^ streptomycin. The endogenous Na^+^ content of the medium was 11 mM as determined by atomic absorption spectroscopy [17]. If indicated, D,L-lactate (33 mM final concentration) was added from a stock solution adjusted to pH 6.5 with KOH. To inoculate the swarming plates, overnight cultures of the Δ*nqrA-F* mutant strain, its parent strain or the non-motile *V. cholerae* O395-N1 Δ*pomAB* [13] were diluted in LB medium to a starting OD600 of 0.01. Cells were grown until OD600 of 0.5–0.6 was reached and washed in fresh LB medium. Cell suspensions were adjusted to OD600 of 0.5 with LB, and aliquots of 1.5 µl were spotted on dried soft agar plates. The diameters of swarming diameters were determined after 16 h at 37°C. Mean values and standard error from 16 experiments are presented. P values were calculated using Student's t test.

## Results and Discussion

### The Δ*nqrA-F* mutant strain showed multiple defects in metabolism detected by the phenotype microarray

We had previously performed a Phenotype Microarray (Biolog) analysis to address how a Δ*nqrA-F* mutation affects *V. cholerae* metabolism and changes in metabolic profiles were determined for the *V. cholerae* parent strain and the Δ*nqrA-F* mutant strain [10]. We had found that the Δ*nqrA-F* mutant has defects in the utilization of several TCA cycle intermediates (fumaric acid, succinic acid and L-malic acid) and many carbon sources that are metabolized into TCA cycle intermediates (L-aspartic acid, L-histidine, L-glutamine and L- glutamic acid). However, because succinic acid was used as the carbon source in the PM3-PM8 plates (nitrogen source, phosphorus source, sulfur sources and nutrient supplements testing plates), the systematic metabolic defects observed in the Δ*nqrA-F* mutant and effects of Δ*nqrA-F* mutation on these metabolisms were still uncertain. Our previous Phenotype Microarray results indicated that the Δ*nqrA-F* mutant utilizes pyruvate as the carbon source similar to the parent strain. Consistent with this, the Δ*nqrA-F* mutant grew similar to the parent strain when grown in M9 minimal media supplemented with pyruvate as the sole carbon source (data not shown). Thus, we tested the PM3-PM8 again using pyruvate as a sole carbon source. The results of the phenotype microarray indicated that the *V. cholerae* O395N1 Δ*nqrA-F* mutant strain still showed multiple defects in nitrogen, phosphate and sulfur utilization (Table S1). Using PM 9 and PM 10 plates, we also tested the effects of osmolytes and pH and found that the Δ*nqrA-F* mutant is sensitive to sodium chloride, sodium sulfate, ethylene glycol, and urea (Table S1). These data again confirmed that lack of Na^+^-NQR broadly affects *V. cholerae* metabolism.

### Transcriptome analyses

We next performed DNA microarray analyses to investigate how the lack of Na^+^-NQR broadly affects gene expression pattern in *V. cholerae*. We had previously found that the Δ*nqrA-F* mutant increased virulence gene expressions, including *toxT*, *ctxB* and *tcpA*, but only at the early phase of the logarithmic growth [18]. This finding suggested that the lack of Na^+^-NQR affected *V. cholerae* gene expressions differently at different phases of growth. Because we aimed to understand the overall changes of gene expression pattern in the Δ*nqrA-F* mutant strain, we performed microarray analyses using RNA prepared from bacterial cultures both at the early and late phases of the logarithmic growth. At the early logarithmic growth, 612 genes were up-regulated and 660 genes were down-regulated in the Δ*nqrA-F* mutant compared to the parent strain, whereas at the late logarithmic growth phase, 119 genes were up-regulated and 264 genes were down-regulated in the Δ*nqrA-F* mutant (data not shown). Consistent with our previous data, virulence genes, including *toxT*, *tcpA-F* and *ctxB* were up-regulated in the Δ*nqrA-F* mutant at the early logarithmic growth phase but not at the late logarithmic growth phase (data not shown). Among these changes, 31 genes were commonly up-regulated and 55 genes were commonly down-regulated in both growth phases (Table 1 and 2).

**Table 1:** Genes up-regulated in the *ΔnqrA-F* mutant based on microarray analysis.

VC number	Functions	Fold change (early)	Fold change (mid)
VC0280	lysine/cadaverine antiporter, cadB	17.836 up	8.313 up
VC0281	lysine decarboxylase, cadA	26.554 up	4.988 up
VC0479	hypothetical protein	2.075 up	1.516 up
VC0615	endoglucanase-related protein	1.709 up	1.568 up
VC0620	peptide ABC transporter, periplasmic peptide-binding protein	1.686 up	2.131 up
VC0786	D-amino acid dehydrogenase small subunit	2.512 up	1.881 up
VC1203	urocanate hydratase	3.598 up	2.000 up
VC1204	formimidoylglutamase	3.152 up	2.211 up
VC1205	imidazolonepropionase	2.674 up	2.062 up
VC1480	hypothetical protein	1.677 up	1.811 up
VC1481	hypothetical protein	1.654 up	1.640 up
VC1627	pH-dependent sodium/proton antiporter, nhaA	2.199 up	2.089 up
VC1689	hypothetical protein	2.292 up	1.634 up
VC1752	hypothetical protein	1.611 up	1.592 up
VC1827	mannose-6-phosphate isomerase	6.706 up	1.662 up
VC1828	hypothetical protein	2.703 up	1.629 up
VC2216	hypothetical protein	2.216 up	1.527 up
VC2361	autonomous glycyl radical cofactor GrcA	2.197 up	1.920 up
VC2556	hypothetical protein	1.572 up	1.519 up
VC2699	anaerobic C4-dicarboxylate transporter	3.431 up	1.509 up
VCA0029	transcriptional regulator, putative	3.633 up	2.241 up
VCA0562	hypothetical protein	1.674 up	1.585 up
VCA0702	iron-containing alcohol dehydrogenase	1.770 up	1.841 up
VCA0732	hypothetical protein	2.365 up	1.746 up
VCA0744	glycerol kinase	1.653 up	2.770 up
VCA0773	methyl-accepting chemotaxis protein	2.453 up	1.937 up
VCA0811	N-acetylglucosamine-binding protein A	7.632 up	2.174 up
VCA0827	pterin-4-alpha-carbinolamine dehydratase	1.542 up	2.797 up
VCA0948	hypothetical protein	2.456 up	1.554 up
VCA1045	PTS system, mannitol-specific IIABC component	1.732 up	2.479 up
VCA1046	mannitol-1-phosphate 5-dehydrogenase	1.997 up	1.712 up

**Table 2:** Genes down-regulated in the Δ*nqrA-F* mutant based on microarray analysis.

VC number	Functions	Fold change (early)	Fold change (mid)
VC0022	hypothetical protein	1.657 down	1.644 down
VC0061	thiamine biosynthesis protein ThiC	1.916 down	1.739 down
VC0062	thiamine-phosphate pyrophosphorylase	1.935 down	1.724 down
VC0063	thiF protein	1.730 down	1.864 down
VC0302	putative 3-phenylpropionic acid transporter	2.403 down	1.821 down
VC0730	copper homeostasis protein	1.675 down	1.647 down
VC0734	malate synthase	2.689 down	1.789 down
VC0751	co-chaperone HscB	1.651 down	1.828 down
VC0754	hypothetical protein	1.899 down	1.755 down
VC0766	exodeoxyribonuclease VII large subunit	1.792 down	2.076 down
VC0769	chitinase, putative	2.752 down	1.532 down
VC0916	phosphotyrosine protein phosphatase	3.258 down	1.622 down
VC0917	UDP-N-acetylglucosamine 2-epimerase	2.858 down	2.191 down
VC1070	phosphatase, putative	1.752 down	1.591 down
VC1124	hypothetical protein	1.561 down	1.519 down
VC1267	hypothetical protein	1.761 down	1.515 down
VC1312	alanine racemase	1.625 down	1.767 down
VC1454	RstA1 protein	3.940 down	1.617 down
VC1461	colonization factor	2.246 down	2.188 down
VC1777	sialic acid-specific TRAP transporter, SiaP	2.354 down	1.992 down
VC1778	sialic acid-specific TRAP transporter, SiaQ	3.571 down	1.923 down
VC1779	sialic acid-specific TRAP transporter, SiaM	3.150 down	2.187 down
VC1782	N-acetylmannosamine kinase	7.239 down	1.986 down
VC1783	N-acetylglucosamine-6-phosphate deacetylase	5.481 down	1.767 down
VC1784	neuraminidase	2.475 down	2.646 down
VC1927	C4-dicarboxylate transport protein	1.745 down	1.763 down
VC1928	C4-dicarboxylate transport protein DctQ, putative	1.970 down	1.947 down
VC1929	C4-dicarboxylate-binding periplasmic protein	2.449 down	2.796 down
VC2037	Na^+^/H^+^ antiporter, nhaC-1	1.680 down	1.599 down
VC2127	flagellar basal body-associated protein FliL	1.885 down	1.602 down
VC2128	flagellar hook-length control protein FliK, putative	4.759 down	1.826 down
VC2130	flagellum-specific ATP synthase	2.060 down	1.915 down
VC2131	flagellar assembly protein H	1.807 down	1.805 down
VC2132	flagellar motor switch protein G	1.519 down	1.663 down
VC2133	flagellar MS-ring protein	1.654 down	1.547 down
VC2136	sensory box sensor histidine kinase	1.682 down	1.533 down
VC2140	flagellar capping protein	1.824 down	1.562 down
VC2141	flagellar protein FlaG	1.914 down	1.611 down
VC2187	flagellin	1.617 down	1.554 down
VC2190	flagellar hook-associated protein FlgL	5.302 down	1.728 down
VC2192	peptidoglycan hydrolase	5.239 down	1.591 down
VC2195	flagellar basal body rod protein FlgG	4.720 down	1.538 down
VC2197	flagellar hook protein FlgE	2.561 down	1.514 down
VC2705	sodium/solute symporter, putative	4.691 down	1.963 down
VCA0176	methyl-accepting chemotaxis protein	2.111 down	1.667 down
VCA0186	hypothetical protein	3.613 down	1.670 down
VCA0204	ATP-dependent RNA helicase RhlE	1.708 down	1.677 down
VCA0699	glucose-1-phosphate adenylyltransferase	1.700 down	1.620 down
VCA0700	chitodextrinase	4.589 down	1.619 down
VCA0835	hypothetical protein	1.876 down	1.611 down
VCA0836	hexapeptide repeat-containing acetyltransferase	1.722 down	1.679 down
VCA0847	inner membrane protein YjeH	2.362 down	1.638 down
VCA0848	GGDEF family protein	2.354 down	1.502 down
VCA0862	long-chain fatty acid transport protein	4.995 down	1.830 down
VCA0864	methyl-accepting chemotaxis protein	1.582 down	1.694 down

The *cadBA* genes showed the highest increases in gene expression levels in the Δ*nqrA-F* mutant in both growth phases (Table 1). The *cadA* gene encodes a lysine decarboxylase and *cadB* encodes a lysine/cadaverine antiporter. The expression of *cadBA* is regulated by a ToxR-type transcriptional regulator, CadC [19], and a LysR type transcriptional regulator, AphB [20]. However, expression of the other AphB regulated genes, such as *tcpP* and *nhaB* [20], were not affected by the Δ*nqrA-F* mutation. Thus, CadC might be responsible for the induced expression of *cadAB* in response to lack of Na^+^-NQR. CadC is known to induce *cadAB* expression in response to acidic pH [19] and we previously found that the *V. cholerae* Δ*nqrA-F* mutant showed increased acetate production and caused acidification of the external medium [10]. Thus, it is likely that *cadAB* expression was induced by the acidic pH that results when Na^+^-NQR function is impaired.

Several transporter genes including tripartite ATP-independent periplasmic (TRAP) transporters, *siaPQM* and *dctMQP*, were down-regulated in the Δ*nqrA-F* mutant strain compared to the parent strain (Table 2). It was recently reported that SiaPQM is a Na^+^-dependent sialic acid-specific TRAP transporter [21], [22]. Consistent with this, multiple genes encoding enzymes in the sialic acid utilization pathway, which convert sialic acid to fructose 6-phosphate, and neuraminidase, which convert host cell surface polysialogangliosides to GM1 monoganglioside and release sialic acid [23], were also down-regulated in the Δ*nqrA-F* mutant (Table 2). Given that Na^+^-NQR and sialic acid catabolic pathways are essential for *V. cholerae* colonization in the small intestine of mice [11], [24], such decreased expression of genes in the sialic acid utilization pathway might explain why the Δ*nqrA-F* mutant showed defects in colonization in the small intestine of mice. The *dctMQP* genes were recently shown to encode a C4-dicarboxylate-specific TRAP transporter and to be partly responsible for *V. cholerae* C4-dicarboxylates, succinate, malate and fumarate, utilization [21]. Thus, it might be possible that the decreased utilization of succinate, malate and fumarate by the *V. cholerae* Δ*nqrA-F* mutant [10] was simply caused by the decreased uptakes of these C4-dicarboxylates. Further studies to better investigate the intriguing links between gene regulation events in response to loss of Na^+^-NQR and changes in *V. cholerae* carbon utilization are required to better understand these observations.

We also found that two methyl-accepting chemotaxis proteins (MCPs), encoded by VCA0176 and VCA0864, were down-regulated in the *V. cholerae* Δ*nqrA-F* mutant compared to the parent strain (Table 2). Interestingly, we found that a VCA0864 mutant strain of *V. cholerae* C6706 showed a hypermotile phenotype in minimal media supplemented with N-acetylglucosamine chemotaxis plate (unpublished data). Together with the fact that the sialic acid degradation pathway and transport system were down-regulated in the *V. cholerae* Δ*nqrA-F* mutant, these data suggest that the *ΔnqrA-F* mutant might be sialic acids starved.

The Class II, III and IV flagellar genes were systematically down regulated in the *ΔnqrA-F* mutant compared to the parent strain (Table 2). One might speculate that the decreased amount of regulatory and structural components of the polar flagellum might result in diminished motility of the Δ*nqrA-F* mutant strain. Indeed, the diameter of its swarming rings on soft agar plates (pH 6.5) reached only 80% of the diameter observed with the parent strain, but was clearly motile when compared with the non-motile Δ*pomAB V. cholerae* strain lacking essential components of the flagellar stator.

**Figure 1:**
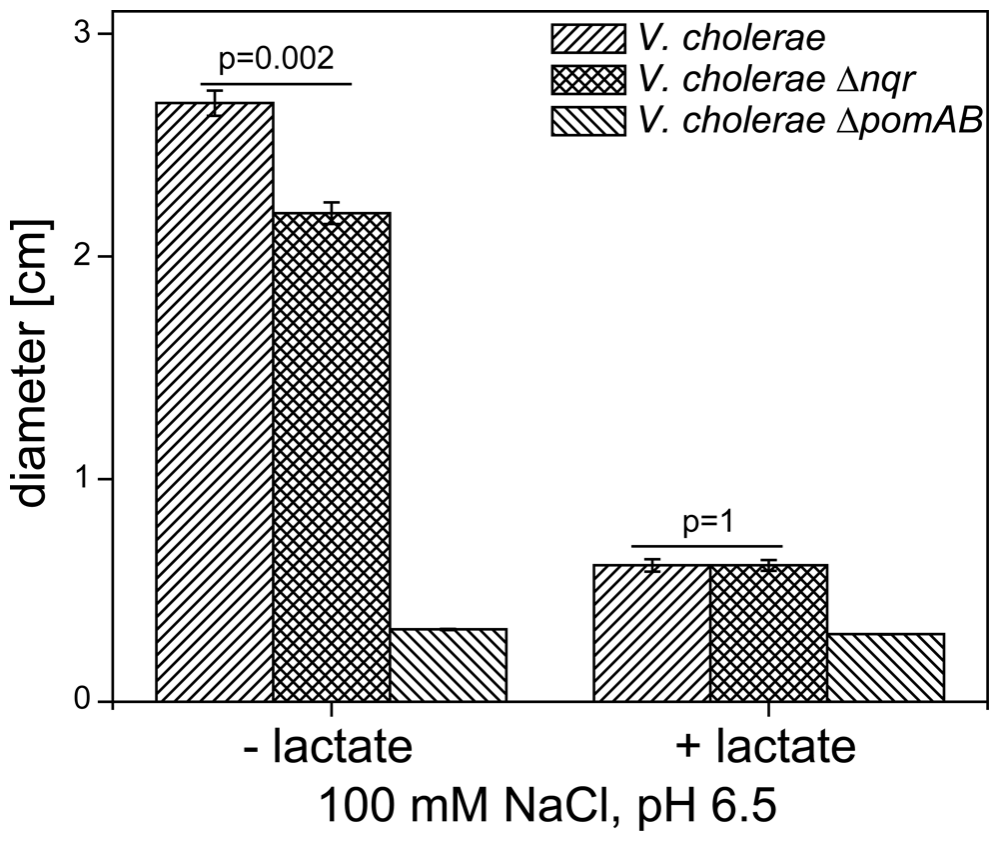
Effect of Δ*nqrA-F* mutation on swarming activity. Swarming assays were performed in LB medium supplemented with 100 mM NaCl and buffered to pH6.5 either with or without the addition of 33 mM D, L-lactate. Mean values and standard error from 16 experiments are presented. P values were calculated using Student's t test.

When lactate was added, both parent and mutant strain exhibited diminished swarming, and the improved motility of the parent strain was no longer apparent (p  =  1, Fig.1). This indicates that depending on the external medium, the mutant strain suffers from a limitation in energy supply for flagellar rotation, resulting in diminished motility.

### Metabolomic analyses

To further understand how lack of Na^+^-NQR affects *V. cholerae* metabolism, we performed a mass spectrometry based metabolomic profiling. We found that 12 metabolites were significantly decreased and 3 metabolites were increased in the *V. cholerae* Δ*nqrA-F* mutant compared to the parent strain (Table 3).

**Table 3:** Metabolites changed in the Δ*nqrA-F* mutant based on metabolomics analysis.

Metabolite	Fold change (nqr/WT)	p-value (t-test)
Adenine	0.737	0.0246
Adenosine	0.248	0.0299
AMP	1.105	0.6629
Arginine	0.964	0.4638
Asparagine	0.936	0.1727
Cadaverine	2.400	< 0.0001
Cyclic AMP	0.095	0.0066
Cyclic GMP	0.094	0.0035
Deoxyribose	0.769	0.1178
dGMP	0.260	0.0025
Fructose-1,6-bisphosphate	0.772	0.2946
Glucose	0.795	0.1613
Glutamate	0.875	0.0220
GMP	∼ 0.10	N/A
Guanine	0.772	0.0065
Guanosine	0.758	0.0056
Histidine	0.956	0.4699
Hypoxanthine	1.411	0.0222
Inosine	0.594	0.0003
Iso/citrate	0.756	0.0027
Isoleucine	0.927	0.1445
Lactate	0.739	0.0024
Lysine	0.596	0.0004
Malate	1.809	0.0003
Methionine	0.873	0.1405
Phenylalanine	0.886	0.0961
Phosphoglycerate	1.094	0.7458
Proline	0.896	0.2215
Ribose phosphate	0.821	0.1549
Serine	0.899	0.1519
Succinate	1.664	0.0934
Threonine	0.898	0.0837
Tryptophan	0.818	0.0599
Tyrosine	0.866	0.1397
Uracil	1.461	0.0010
Valine	0.861	0.1073

**Figure 2:**
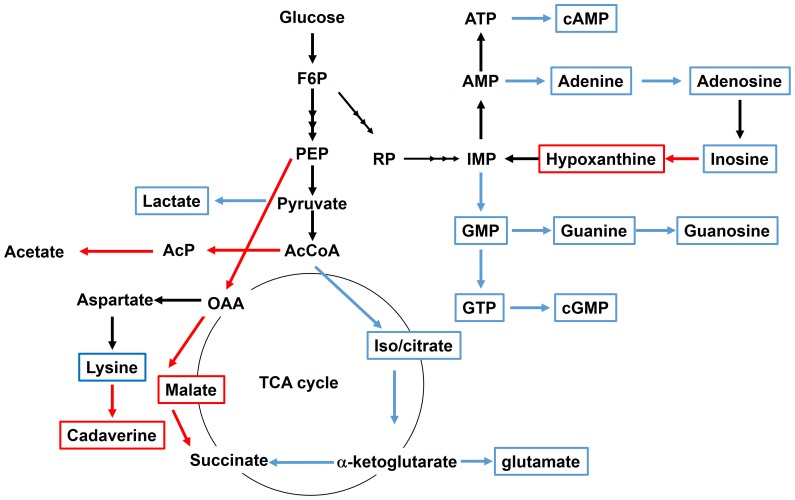
Changes in central metabolism in *V. cholerae* Δ*nqrA-F* mutant. Red solid squares show metabolites that are increased in the Δ*nqrA-F* mutant. Blue solid squares show metabolites that are decreased in the Δ*nqrA-F* mutant. Red solid arrows show metabolic pathways that are expected to be decreased in the Δ*nqrA-F* mutant. Blue solid arrows show metabolic pathways that are expected to be increased in the Δ*nqrA-F* mutant. AcP, acetyl phosphate. RP, Ribose phosphate.

We had previously suggested that TCA cycle activity is decreased in the *V. cholerae* Δ*nqrA-F* mutant [10]. Consistent with this, intracellular isocitrate levels were decreased in the Δ*nqrA-F* mutant (Table 3). However, we unexpectedly found that intracellular malate levels were significantly increased in the Δ*nqrA-F* mutant (Table 3). We also detected slightly increased levels of succinate in the Δ*nqrA-F* mutant (data not shown). Taken together, this may suggest that the reductive pathway of the TCA cycle is more active in the *V. cholerae* Δ*nqrA-F* mutant (Fig. 2).

We found that intracellular lysine levels were decreased, while cadaverine levels were increased in the *V. cholerae* Δ*nqrA-F* mutant compared to the parent strain (Table 3). Since CadA catalyzes the conversion of lysine to cadaverine, these changes could be caused by the increased *cadA* expression that we detected in the transcriptome analyses.

### The acetate switch is broken in the *V. cholerae* Δ*nqrA-F* mutant

**Figure 3:**
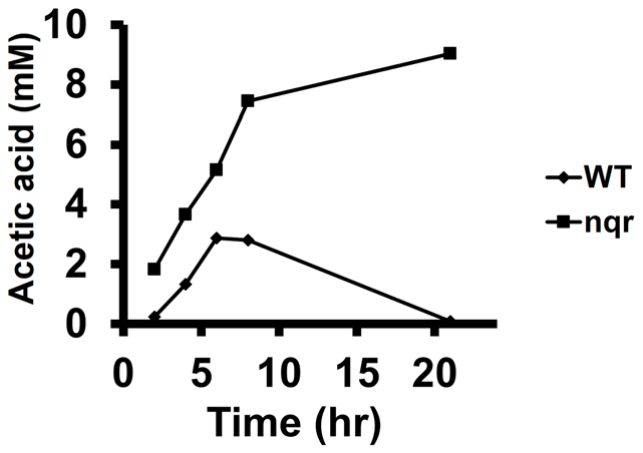
Effect of Δ*nqrA-F* mutation on acetate secretion. *V. cholerae* O395N1 and *V. cholerae* O395N1 Δ*nqrA-F* strains were inoculated into LB (pH 6.5) at 30°C. Acetic acids levels in the medium were measured using the acetic acid enzymatic assay kit (R-Biopharm).

We had previously shown that the *V. cholerae* Δ*nqrA-F* mutant showed increased acetate production compared to the parent strain [10]. When grown in tryptone-based rich media, *E. coli* first produces acetate by using the PTA-ACK pathway but at a certain point in its growth phase, it shifts to utilize the excreted acetate by using the AMP forming acetyl-CoA synthetase (AMP-ACS). This transition from acetate production to acetate utilization is called the acetate switch [25]. To further examine acetate production in the *V. cholerae* Δ*nqrA-F* mutant, we measured a time course of acetate excretion. Between 6 hr and 8 hr of growth in LB media, the *V. cholerae* parent strain started to reduce external acetate production, indicating that *V. cholerae* also has an acetate switch (Fig. 2). Interestingly, the *V. cholerae* Δ*nqrA-F* mutant did not show this phenotype and kept excreting acetate until the end of growth (Fig. 3). This is consistent with the Phenotype Microarray data that showed that the *V. cholerae* Δ*nqrA-F* mutant had a defect in acetate utilization (Table S1). The AMP-ACS protein is known to be regulated by protein acetylation with the acetylated form of AMP-ACS being inactive and these defects in acetate utilization in the Δ*nqrA-F* mutant suggested that AMP-ACS might be acetylated.

### The Δ*nqrA-F* mutation does not affect osmotic resistance in V. cholerae

The results of the phenotype microarrays showed that the *V. cholerae* Δ*nqrA-F* mutant strain was sensitive to osmotic stress including NaCl and ethylene glycol (Table S1). Thus, we performed growth assays in LBB-based medium (noncationic L broth), containing increasing concentrations of NaCl and ethylene glycol. Consistent with the phenotype microarrays, the *V. cholerae* Δ*nqrA-F* mutant showed growth defect compared with the parent strain but the growth defect was independent to the increasing concentrations of NaCl (Quinn et. al. submitted) or ethylene glycol (data not shown). To further determine whether the effect of Na^+^-NQR on growth was primarily related to loss of quinone reduction or more general Na^+^ homeostasis, L-lactate was added to the growth media, which is expected to allow the L-lactate dehydrogenase to replenish the quinone pool directly. Interestingly, addition of L-lactate restored the growth of the *V. cholerae* Δ*nqrA-F* mutant to the parent level even in the presence of high concentration of NaCl (Quinn et.al. submitted) or ethylene glycol (data not shown). These data indicated that lack of Na^+^-NQR does not directly affect osmotic stress resistance in *V. cholerae*. Our transcriptome analyses detected the increased expression of a major Na^+^/H^+^ antiporter gene, *nhaA* in the *V. cholerae* Δ*nqrA-F* mutant (Table 1), suggesting that NhaA might complement the Na^+^ pumping activity of Na^+^-NQR.

## Conclusions

Lack of Na^+^-NQR broadly affects *V. cholerae* physiology but mainly affects its central metabolism but not Na^+^ pumping-related phenotypes.

## Supporting Information

Table S1 **Phenotypes of the V. cholerae Δ** ***nqrA-F*** **mutant based on Biolog phenotype microarrays.**
(XLSX)
